# Supplementary material for: Reconciling Mining with the Conservation of Cave Biodiversity: A Quantitative Baseline to Help Establish Conservation Priorities
Source: PLoS One. 2016 Dec 20;11(12):e0168348. doi: 10.1371/journal.pone.0168348 (PMC5173368; doi:10.1371/journal.pone.0168348)
Supplement: S1 Dataset — (ZIP) [file pone.0168348.s002.zip › Taxa/Serra Norte/SN_2013/N4E/Lista N4E-64.pdf]

CAVIDADE N4E-0064

Classe	Ordem	Fam/Outros	Gên/Outros	Espécie	S-U	U-S	Zon.
Annelida	Oligochaeta			jovem		2	E/P
Arachnida	Acari	(Parasitengonina)		jovem		X	E
Arachnida	Acari	Gamasida		jovem		X	P
Arachnida	Acari	Laelapidae		sp.77	X		P
Arachnida	Acari	Prostigmata		jovem	X		E
Arachnida	Acari	Rhagidiidae		sp.20	X		P
Arachnida	Amblypygi	Phrynidae	*Heterophrynus*	*longicornis*	3	1	E/P
Arachnida	Araneae	Ctenidae	*Ancylometes*	*rufus*	2	2	E
Arachnida	Araneae	Pholcidae	*Mesabolivar*	*aurantiacus*	X	X	E
Arachnida	Araneae	Pholcidae	*Mesabolivar*	sp.1	X		E
Arachnida	Araneae	Pisauridae		jovem	5		E
Arachnida	Araneae	Salticidae		sp.2	X	X	E
Arachnida	Araneae	Scytodidae	*Scytodes*	*eleonorae*	1		E
Arachnida	Araneae	Theridiosomatidae	*Natlo*	sp.1		X	E
Arachnida	Araneae	Theridiosomatidae	*Ogulnius*	sp.1		X	E
Arachnida	Araneae	Theridiosomatidae	*Plato*	sp.1	X	X	E/P
Arachnida	Opiliones	Cosmetidae	*Roquettea*	*singularis*	2		E
Arachnida	Opiliones	Sclerosomatidae	*Geaya*	sp.1	2		E
Arachnida	Opiliones	Sclerosomatidae	*Prionostema*	sp.1	2	3	E/P
Arachnida	Pseudoscorpiones			jovem	X		P
Chilopoda	Scutigeromorpha	Pselliodidae		jovem	1		E
Diplopoda	Polydesmida	Cyrtodesmidae		sp.1	X		E
Entognatha	Collembola	Entomobryidae		sp.7		X	E/P
Entognatha	Collembola	Isotomidae		sp.1	X		P
Entognatha	Collembola	Paronellidae		sp.1	X	X	E/P
Entognatha	Collembola	Paronellidae		sp.3		X	P
Entognatha	Collembola	Paronellidae		sp.4	X		P
Entognatha	Diplura	Campodeidae		sp.1	X		E
Insecta	Blattodea			jovem	1		E
Insecta	Coleoptera	Carabidae		sp.4	X	X	E
Insecta	Coleoptera	Elateridae		sp.3	X		E
Insecta	Coleoptera	Staphylinidae	Pselaphinae	sp.2	X	X	E/P
Insecta	Coleoptera	Staphylinidae	Pselaphinae	sp.21	X		E
Insecta	Coleoptera	Staphylinidae	Pselaphinae	sp.31	X		P
Insecta	Coleoptera	Staphylinidae		sp.33		X	P
Insecta	Coleoptera	Staphylinidae		sp.49	X		E
Insecta	Diptera	Chaoboridae		sp.	X		P
Insecta	Diptera	Culicidae	*Culex*	sp.	X		E/P
Insecta	Diptera	Dolichopodidae		sp.	X		E
Insecta	Diptera	Phoridae	Metopininae	sp.		X	P
Insecta	Diptera	Sciaridae		sp.	X		E/P
Insecta	Diptera	Tipulidae	Tipulinae	sp.	X	X	E/P
Insecta	Homoptera	Cixiidae		sp.3	X		P
Insecta	Hymenoptera	Formicidae	*Gnamptogenys*	sp.1	X		E
Insecta	Hymenoptera	Formicidae	*Neivamyrmex*	sp.1	X		P
Insecta	Hymenoptera	Formicidae	*Pachycondyla*	sp.1	X	X	E/P
Insecta	Hymenoptera	Formicidae	*Pachycondyla*	sp.2	X		E/P
Insecta	Hymenoptera	Formicidae	*Solenopsis*	sp.1		X	P
Insecta	Isoptera	Termitidae	*Nasutitermes*	sp.	X		E/P

Insecta	Lepidoptera	Noctuoidea		sp.7	X	1	E
Insecta	Orthoptera	Phalangopsidae	*Paraclodes*	sp.	3		E
Insecta	Orthoptera	Phalangopsidae	*Phalangopsis*	jovem	9		E/P
Insecta	Psocoptera	Epipsocidae	*Epipsocus*	sp.2	X		E
Malacostraca	Decapoda			sp.	1		E
Malacostraca	Isopoda	Dubioniscidae		sp.1	X		E
Symphyla		Scutigerellidae		sp.	X		P
Amphibia	Anura	Strabomantidae	*Pristimantis*	cf. *fenestratus*	8		E/P
Mammalia	Chiroptera	Emballonuridae	*Peropteryx*	*kappleri*	3	2	E/P
Mammalia	Chiroptera	Furipteridae	*Furipterus*	*horrens*	2		P
Mammalia	Rodentia	Cricetidae	*Rhipidomys*	sp.	2		E/P
